# The Experiences of People With Mild Traumatic Brain Injury Attending Emergency Departments in England

**DOI:** 10.1097/HTR.0000000000001168

**Published:** 2026-04-23

**Authors:** Faye Forsyth, Anne Manktelow, Daniel Whitehouse, Susie Hardwick, Kerry Meynell, Graham Martin, Virginia F. J. Newcombe

**Affiliations:** ***Author Affiliations:*** *Primary Care Unit (Forsyth), Department of Public Health & Primary Care, University of Cambridge, Cambridge, United Kingdom; KU Leuven Department of Public Health and Primary Care (Forsyth), KU Leuven, Leuven, Belgium; PACE Section (Perioperative, Acute, Critical Care and Emergency Medicine) (Forsyth, Manktelow, Whitehouse, and Newcombe), Department of Medicine, University of Cambridge, Cambridge, United Kingdom; EURECA (Emergency and Urgent Care Research Cambridge) (Whitehouse and Newcombe), PACE Section, Department of Medicine, University of Cambridge, Cambridge, United Kingdom; Emergency Department (Whitehouse, Hardwick, Meynell, and Newcombe), Addenbrooke's Hospital, Cambridge University Hospitals NHS Foundation Trust, Cambridge Biomedical Campus, Cambridge, United Kingdom; The Healthcare Improvement Studies Institute (Martin), Strangeways Research Laboratory, Cambridge, United Kingdom.*

**Keywords:** mild traumatic brain injury, patient experiences

## Abstract

**Aims:**

To establish the health care management experiences of people who had sustained a mild traumatic brain injury (mTBI) in the United Kingdom.

**Methods:**

Interviews with people who had recently sustained a mTBI in the United Kingdom were conducted online using the platform Microsoft Teams and simultaneously digitally recorded and auto transcribed. Transcripts were cleaned and checked for accuracy before being subjected to inductive thematic analysis following the 6 phases outlined by Braun and Clarke.

**Results:**

Seventeen people with experience of mTBI and one carer participated in this study (n = 18 interviews). Three broad themes with 9 subthemes were generated. Themes mapped to the mTBI patient's health care journey accessing (prehospital), traversing (in-hospital) and exiting health care services (posthospital). Prehospital experiences were characterized by “*Decisional Difficulty*” (Theme 1); notably, there were challenges deciding whether symptoms were the result of an mTBI or preexisting disease, whether the mTBI was severe enough to warrant medical review, and which service was most appropriate to present to. In-hospital care was described as “Lacking Holism” (Theme 2) and participants voiced that there was a focus on the injury, not the person. Care was experienced as fragmented and discharge information and follow-up were frequently described as an afterthought. The posthospital recovery landscape was depicted as “*Uncertainty Central*” (Theme 3); uncertainties arose in part from the lack of information on recovery trajectories provided at discharge. Participants also described a desire for reassurance to help them navigate these uncertainties, and the need for work and home spaces that are sensitized to recovery.

**Conclusions:**

The label mTBI has long been recognized as a misnomer given the significant debilitating impacts it can have in both the short and long term. This research describes patients' experiences of mTBI from a health care management perspective. It illuminates the suboptimal care pathways and management processes experienced by many. While there was staunch sympathy for health care providers and acknowledgement of the significant pressures they face, there is nevertheless a need for significant changes across primary, secondary, and tertiary care to ensure that the informational and care needs of people with mTBI are met. Greater public awareness in mTBI is also required, so that family members and other significant parties realize the scope of the impact. Finally, employment policies need to change to ensure that people with mTBI are supported in their recovery.

## Abstract 2

Supplemental Digital Content is Available in the Text.

## INTRODUCTION

Mild traumatic brain injury (mTBI), which may be defined as transient loss of consciousness, a Glasgow Coma Score of 13–15, presence of post-traumatic amnesia, focal neurological deficits and/or an alteration of mental state, is very common.^1^ Worldwide it is estimated that 50–60 million people sustain a traumatic brain injury each year, with the majority (80%–90%) being classified as mild.^2^ It has generally been assumed that most people who sustain a mTBI would recover completely within the first few weeks of injury.^1,3^ As a result, management within the emergency department (ED) is focused upon ruling out serious injuries that may require neurosurgical intervention and/or observation, and there is much less emphasis on any longer-term sequelae.^2,4^ The label “mild,” however, is misleading.^5^ Two large multicenter studies have shown that postconcussive symptoms (PCS), a cluster of somatic, cognitive, and emotional symptoms,^6^ persist in approximately half of patients for greater than 12 months after mTBI.^7,8^ Symptom burden is varied; however, many describe substantial disability including cognitive, psychological, and somatic problems that severely impacts quality of life. Indeed, as a result of mTBI, patients may experience relationship difficulties,^9^ increased health care utilization,^10,11^ depression,^12^ employment difficulties, and other consequences that may last many years after initial injury.^13,14^

There is low-certainty evidence that early advice and educational material may improve outcomes.^15,16^ This forms the basis of recommendations within the UK National Institute for Health and Care Excellence (NICE) Guidelines for Early Management of Head Injury [NG232].^2^ However, these recommendations are inconsistently applied. One UK study suggested that as few as 14% of patients had a description within their clinical record evidencing advice provision; when it was given, it lacked standardization.^17^ Other research has determined that appropriate follow-up care, commensurate with the severity of reported symptoms, is often lacking.^18^ There is no formal pathway for the management of mTBI in the United Kingdom,^19^ and for many patients, particularly those with normal computed tomography head scans, the ED may be their only route of access to care and/or route to onward referrals.^20^

## BACKGROUND

While there are qualitative studies in patients with severe traumatic brain injury or mixed severity populations,^21–23^ there is a paucity of studies focused specifically on mTBI. Furthermore, most studies have focused on the downstream effects of mTBI. As a result, there is limited understanding of how acute care is perceived and how it may influence recovery. The research presented here sought to address this research gap by establishing the health care management experiences of people who had sustained a mTBI (in the acute or subacute phase) and subsequently sought medical review from an ED within the United Kingdom. The objectives were to (1) understand their clinical trajectories, (2) collate their experiences of medical care, and (3) establish their views on recovery and barriers and facilitators to recovery.

## METHODS

Findings are reported in line with the COREQ checklist (consolidated criteria for reporting qualitative research).^24^ Ethical approval was granted by the North of Scotland Research Ethics Committee 1 (REC Reference 23/NS/0141). Informed consent was obtained from all participants.

## RESEARCH TEAM AND REFLEXIVITY

### Personal characteristics

Interviews were conducted by 2 female authors (A.M., V.F.J.N.), the study coordinator (Psychologist with experience of mTBI assessment and follow-up) and Principal Investigator (Consultant in Emergency Medicine and Intensive Care), respectively. The transcripts were analyzed by one female researcher (F.F.) with qualitative expertise. All researchers had an extensive background in mTBI research (range 8–20+ years) and were experienced in conducting clinical assessments and research interviews in mTBI. V.F.J.N. had active clinical responsibility for managing mTBI in the ED and has contributed to clinical guidelines on mTBI. Both interviewers and the coder approached the project with the assumption that mTBI care is inadequate and a belief that a change in practice is needed. These assumptions and biases may have influenced or affected responses.

For example, the semistructured interview schedules were focused upon the clinical pathway and problems experienced. Responses provided by interviewees were often followed up with probing questions about the acceptability or quality of care received; these orientations may have influenced the feedback obtained. Transcripts were coded by a researcher who was not involved in the interview process, however with a similar background in mTBI research. As a result, the analysis may have foregrounded care pathway deficiencies, potentially at the expense of other important insights. To counterbalance this potential bias, a broad sampling frame was used, that is, consecutive cases were recruited by research nurses in the ED who were not sensitized in the same way. Furthermore, divergent cases were sought and used as comparisons in the analysis.

### Relationship with participants

During the study, contact with the participants took place during the recruitment process (telephone contact) and at interview. The study coordinator conducted most of the participant scheduling and interviews. All procedures were performed under the oversight of the Principal Investigator. Participants had no prior engagement with the researchers and knew only of their roles and responsibilities through details provided in the participant information sheet and by spontaneous sharing during interviews.

## STUDY DESIGN

### Theoretical framework

This research followed the processes of thematic analysis established by Braun and Clarke.^25^ For 2 reasons in particular, we chose not to deploy an overarching theoretical framework. First, given the novelty of the topic, there was no obvious relevant theory. Second, the interviews were conducted as part of a wider program of work that did not lend itself to a particular theoretical orientation.

### Participant selection

A combination of convenience and purposive sampling was employed. The majority of the participants were approached in the ED of one major trauma center (Addenbrooke's Hospital, Cambridge, United Kingdom) by research staff who introduced the study and gained patients' permission to pass on their details to the study team. The study coordinator reviewed eligibility and contacted potential participants. Six individuals who were willing to discuss the study declined further participation on subsequent contact. We were unable to arrange one interview because of scheduling challenges. A small number of participants (n = 3) self-referred for the study upon encountering study advertisements which were cascaded through professional networks.

Seventeen people with mTBI who were at various stages of their recovery journey and one carer of a person with mTBI were enrolled. The carer was asked to provide demographic details of the person sustaining the injury (included in sample demographics summary), in addition to their own demographic information (not presented). All participants were compensated for their time. Employment data reflect preinjury status; the effects of injury on employment were explored in interview but not documented systematically. Participants had a median age of 52 years (range: 18–85 years) and were predominantly female (n = 12, 67%). Most mTBI participants were within 1 month of injury (n = 11, 61%) and sustained their injury following a fall (n = 10). Full demographic details are provided in Table 1. Interviews lasted a mean of 47 minutes. Participants were predominantly interviewed alone, but on occasion, they were accompanied by (nonparticipating) family members.

**TABLE 1:** Demographic characteristics

Characteristic	Sample
Female sex (n, %)	12 (67)
Age (mean, range)	52 (18–85)
Cause of injury—fall (n, %)	10 (56)
Cause of injury—other nonintentional injury (n, %)	4 (22)
Cause of injury—sport recreation (n, %)	3 (17)
Cause of injury—minor road traffic accident	1 (6)
Months since injury (median, range)	1 (0–24)
White British Ethnicity (n, %)	13 (72)
Married/partner (n, %)	11 (61)
Secondary school level education	5 (28)
Post-secondary school training (trade/technical certificate)	2 (11)
Undergraduate University/college degree/diploma (n, %)	4 (22)
Postgraduate degree education (n, %)	7 (39)
Working 35 hours or more/wk (n, %)	7 (39)
Retired (n, %)	2 (11)
Student (n, %)	2 (11)
Manager/professional (n, %)	7 (39)
Technician/supervisor/associate professional (n, %)	4 (22)
Living with family/friends (n, %)	13 (72)
General Practitioner (GP) follow-up (n, %)	6 (33)
GP follow-up requested by patient (n, %)	6 (100)

### Data collection

A semistructured interview schedule was developed before interviews. The questions were based on the researcher's experience and supplemented with experiential insight from a patient who had sustained a mTBI. The patient was engaged by patient and public involvement work. Questions were not revised; additional prompts were iteratively added based on interview responses. The schedule is available in Supplementary Figure 1, http://links.lww.com/JHTR/B62. All interviews were conducted using Microsoft Teams; video capability was used to facilitate dialog, but only audio records were retained in line with ethical approval. Transcripts were not returned to participants for checking, and repeat interviews were not conducted.

## ANALYZING AND FINDINGS

### Data analysis

Autotranscribed data are subject to a number of vocabulary recognition and grammatical errors^26^; therefore, an extensive process of cleaning and checking was undertaken by (A.M.). Member checking of interview transcripts was not performed for pragmatic and clinical reasons. Interviews were ceased after a defined recruitment period within the ED in line with ethics committee approval and clinical support agreements. This is in line with Braun and Clarke's^27^ approach to thematic analysis which identifies that stopping data collection is “inescapably situated and subjective.” They argue that pragmatic judgments based on time and resource shape sample sizes in qualitative research as they do other types of research and are thus legitimate factors that shape sample size decision-making.^27,28^

One author (F.F.) coded all of the data using Nvivo 12 software^29^; the data analyst had not been involved in this study up to this point. Another author (A.M.) performed inductive coding of one transcript and deductive coding to the framework established by the primary coder (F.F.). There was good alignment between the coding decisions at both inductive and deductive levels. Discrepancies between coding decisions were discussed; these were generally interpretational subtitles, and both coders were comfortable that either interpretation was legitimate.

The analysis followed the 6 phases of thematic analysis described by Braun and Clarke^25^: (1) familiarization, (2) coding, (3) generating themes, (4) reviewing themes, (5) defining and naming themes, and (6) report writing. Familiarization involved reading and re-reading the transcripts. Inductive “data-driven” codes were applied to the entire body of transcripts (line-by-line coding), before codes were grouped into phases corresponding to the person's mTBI health care journey. The next stage involved reviewing the codes, then sorting and collapsing them to form larger potential themes. Coded extracts that represented the initial themes were exported then reviewed again, before decisions were made regarding what constituted a “relevant theme” and what we termed “contextual scaffolding,” that is, information that was relevant to the theme, but which is already understood, like the breadth of post-mTBI symptoms. The remaining themes underwent descriptive refinement, and finalized themes were mapped to form (1) an overarching “journey point” theme (n = 3) and (2) corresponding sub-themes (n = 9).

### Reporting

Three broad themes that corresponded to the 3 main points of the patients' journey through health care services were generated. The prehospital phase was characterized by “*Decisional Difficulty*.” In-hospital care was depicted as “*Lacking Holism*,” and the posthospital phase of a patient's journey was covered under “*Uncertainty Central*.” Themes are discussed in full in the subsequent sections and presented alongside data extracts. Further quotes in support of the themes are presented in Supplementary Table 1, http://links.lww.com/JHTR/B62. The thematic map is presented in Figure 1.

**Figure 1.**
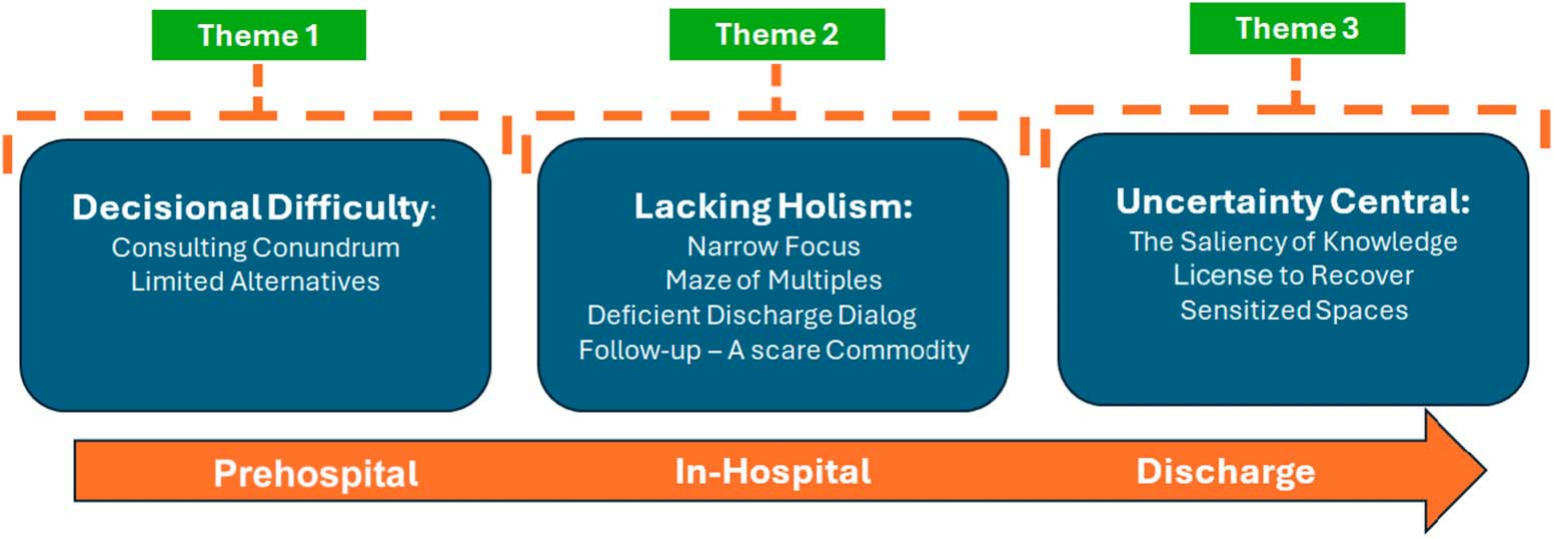
Thematic map.

## DECISIONAL DIFFICULTY

People who were alone when their accident occurred and those with delayed symptom onset faced decision-making challenges. They described negotiating a “Consulting Conundrum” and the “Limited Alternatives” to ED attendance.

### Consulting conundrum

Underlying disease could influence health care access decisions, as participants tried to ascertain whether symptoms experienced were the result of the mTBI or concurrent disease or activities (ie, alcohol consumption). The debate of symptom origin occurred in parallel with an evaluation of severity; both were complicated by ethical questions regarding the justness of attending ED. All 3 considerations (cause, severity, ethics) influenced whether, and when, a person attended the ED. These consulting conundrums were often negotiated in the context of significant post-mTBI side effect like altered brain function (disorientation, inability to concentrate) and physical symptoms like nausea, fatigue, and dizziness, which further hindered decision-making.

“Then a lot of people would think, oh it's ridiculous, I've got a headache, why would I want to go to ED, you know, you can see they're so busy” (IABA011)

### Limited alternatives

Those who decided their mTBI met the ethical or severity threshold warranting medical review, still reported avoiding ED. These individuals sought information online or support from alternative services (general practices, NHS 111). Attempts generally appeared to be thwarted, as robust online self-management or assessment information could not be retrieved, or other services were not available. This resulted in a feeling that there was no alternative but to attend ED.

“…I spent something like 20 minutes on hold waiting for someone to pick up on 111 and there was just no information, not anything available anywhere, which is then why I went to the hospital” (IABA009)

## LACKING HOLISM

People attending ED often perceived there to be a “*narrow focus*” to care. Information was often poorly communicated, as people navigated a “*Maze of Multiples*.” Most participants experienced “*Deficient Discharge Dialogs*” with information often being offered at the last minute and in a rushed fashion. Almost all participants desired further follow-up, but this was described as a “*Scarce Commodity*.”

### Narrow focus

Upon presentation at ED, the focal point of care could be the mTBI, particularly if it was isolated. However, several people reported that care provision could also be anchored to addressing external injuries like head lacerations, or the precipitating cause of any fall related mTBI, to the detriment of mTBI care provision. There appeared to be limited capacity among providers to holistically consider the patient. Some deemed this to be par for the course in ED and acceptable in light of the pressures on services. For others, the narrow focus was experienced as limiting care provision, with one issue being prioritized at the expense of the mTBI management itself.

“they just took it as an old person falling… let's dress his wounds and, you know…take him away from A&E” (IABA016)

### Maze of multiples

The overall picture was of seeing multiple people, in multiple locations, having multiple assessments or clinical procedures, and sometimes receiving multiple (occasionally contradictory) messages on the course of action or outcome. Lack of continuity in care was frequently experienced, and participants described feeling “lost” or “left.” They often evidenced this discontinuity with descriptions of having to seek or provide their own care. A divergent case (n = 1) highlighted the difference patient-centered, attentive care could have.

“You see a dozen people in one form or another…you get the feeling that they're…I wouldn't say they're rushing, but they haven't really got any time to spare” (IABA008)

“I felt I had a Rolls Royce service. I mean, it was really amazing that the doctor we saw really carefully explained the whole process” (IABA024)

### Deficient discharge dialogs

Overall, although there were a handful of satisfactory reports, discharge experiences were described as inadequate. It appeared that whether and how discharge information was provided (verbal, written, quick response [QR] code) was dependent on the clinician. Some people reported leaving with unanswered questions as they were not provided with the opportunity, or appropriate space and time, to ask questions. There were no accounts of tailoring information to the person. When time was taken to comprehensively cover sequelae, management and trajectories at discharge, participants described feeling more satisfied and empowered.

“At the time of the discharge, well, first having a sit-down conversation would have been useful. I remember being stood up in the corridor…shown that QR code; just a sit-down conversation…, maybe in writing?” (IABA002)

### Follow-up—a scarce commodity

Follow-up mTBI care did not frequently feature in participants' stories, which is supported by the quantitative data presented. Although this was understood and accepted to be routine care, there was nonetheless a desire for signposting to follow-up care services or some form of direct follow-up contact. Lack of either type of follow-up led to feelings of being left to “sort yourself out,” or that the “onus is on you” to decide what to do in the face of ongoing symptoms. Some participants were verbally advised to “get in touch” if symptoms developed or persisted, but no contact details were provided.

“but the body functions very differently depending on the environment we evolve in, the constraints we have upon ourselves. So to me, just a quick check 48 hours after to see if everything's OK, and if we need to go back and be seen or have additional support” (IABA002)

## UNCERTAINTY CENTRAL

The impression of existing in “*Uncertainty Central*” was the major theme that encapsulated the postdischarge period. Participants described “*The Saliency of Knowledge*” during this period, and divergent narratives helped unpick the impact of good versus poor knowledge on personal recoveries. Some participants described the need for a medically prescribed “*License to Recover*,” whereby they would like medical permission or reassurance that it is acceptable to take time off from work, or to return to health care services for reassurance and guidance if needed. Recovery was often hampered by a lack of “*Sensitized Spaces*” notably within the home, work, and health care settings.

### The saliency of knowledge

Knowledge of post-mTBI sequelae appeared key to recovery, but because of poor provision at ED discharge, it frequently had to be self-acquired or learned by trial and error. Participants who reported less insight into the potential scale and scope of PCS tended to dive straight back into work and other habitual activities, which had the potential to aggravate symptoms, especially where it involved working with display screens.

“I kind of used some of the information I got off the internet to inform me how I would go back to work…having not had a significant bang to my head before, I just wanted to make sure that what I was feeling was relatively normal” (IABA011)

### A license to recover

There was a strong desire for postdischarge reassurance, guidance, and even a “license” to take recovery time needed. This was particularly important for people who were in employment, as many felt it necessary to return to work within a relatively short time period from injury (1–2 days). Of those who sought follow-up advice from their General Practitioner (n = 6), the desired outcomes were validation that their symptoms were normal, general reassurance or guidance as to progress of their recovery and insights into the duration of lingering symptoms.

“But I guess it's the reassurance for me. It would be the reassurance of it's OK. You're still dizzy, but that's to be expected. It's not unusual. It might have gone by now, but it's not unusual that you've still got it” (IABA036)

### Sensitized spaces

Participants reported significant issues around the “sensitivity” of work and home spaces to aiding recovery. Expectations in both settings could limit the ability to rest (work activities require concentration, children require care and attention). Even employers that one might expect to be informed (NHS), did not appear to be attuned to the implications of PCS in the workplace. A small proportion of participants (n = 2) recounted distressing employer responses like docking pay and emotional blackmail. Some emphasized the importance of having home spaces that were responsive to their recovery needs, and multiple interviewees voiced how valuable it was for their recovery to be able to step back from parental or other home-based duties (cooking, gardening). There was a desire for more information on the return to normal life, even among those with rapid recoveries or minimal symptomology. Within the health care sphere, there appeared to be limited capacity among health care professionals to accommodate mTBI, particularly if symptoms were protracted. The management approach was for further referral to treat PCS as stand-alone complaints, rather than bespoke mTBI management per se.

“And your kind of, you don't wanna say too much to people because you don't wanna come across as being, you know, being difficult in any way and being judged, I suppose. And I think in a way as well when you work for the NHS, you just feel you've got to get on with it” (IABA017)

## DISCUSSION

This analysis has constructed 3 broad themes and 9 subthemes that characterized patients' mTBI management experiences within the NHS in England. Overall care, both direct (NHS services) and indirect (publicly available NHS information), was described as suboptimal. Most participants recognized the significant pressures that EDs are experiencing,^30^ which are driven by an increase in the number of people with complex problems who are visiting the ED, coupled with a lack of onward facilities to support their discharge, or timely follow-up.^19,31^ There was also sympathy and even an expectation that, given this pressure and lack of resource, care might not be optimal. However, many of the substandard experiences reported can be relatively easily addressed through one pathway, upgrading information.

*Theme 1* “*Decisional* *Difficulty*,” which encompassed the sub themes “*Consulting Conundrum*” and “*Limited Alternatives*,” could be tackled by enhancing publicly available information so that it can (1) more effectively support people with mTBI to assess their symptom severity and (2) guide them to the appropriate health care services, which may or may not be the ED. Similarly, many of the issues raised in *Theme 3* “*Uncertainty Central*” which included the subthemes “*Saliency of Knowledge*,” “*License to Recover*” and “*Sensitized Spaces*” could also be addressed by improving the quality and quantity of information about mTBI, the sequelae and potential long-term recovery trajectories. Problems with care described in *Theme 2* “*Lacking Holism*,” may be partly attributed to the systemic pressures faced by EDs that are struggling to deliver basic care in the face of multiple pressures.^30,31^ However, small changes in the way information is communicated could address at least some of the areas identified as being less than ideal in the subthemes “*Maze of Multiples*,” “*Deficient Discharge Dialogs*,” and “*Scarce Commodities*.” Making sure that results are communicated appropriately, consistently supported with postdischarge information and signposting to follow-up avenues, could be a first step. Such suggestions are by no means ambitious and reflect recommendations set out in current NICE guidelines. Further research is required to understand what prevents clinicians from delivering NICE recommendations for mTBI in the ED.

### Comparison with other literature

Multiple small qualitative studies of the mTBI patient experience have been undertaken. Most focus on one component of mTBI like symptoms,^32^ returning to work,^33^ the impact on carers,^34^ or accessing rehabilitation services.^35^ To our knowledge, only 2 qualitative studies have specifically focused on mTBI management from the patient perspective.^22,23^ Linnestad et al^23^ interviewed 8 people post-mTBI (22–55 years) to explore their experiences of injury, treatment, and recovery. They distilled 3 major themes. “Ambiguity and hope” described participants' sense of uncertainty and their search for information and a coherent understanding on their post-mTBI recovery. Snell et al^22^ interviewed 10 people (26–67 years) to explore their experiences of recovery. The overarching sense of the interviews was of trying to achieve a sense of coherence of their injury, symptoms, and recovery.

There are multiple similarities between our study and those of Linnestad et al^23^ and Snell et al.^22^ For example, there is a lack of information and guidance available for people with mTBI, which leads them to feel uncertain about what to do, and unsure whether their recovery trajectory is normal. There is also a need for validation, reassurance, and support in the follow-up period, even in the absence of disruptive symptoms. There are remarkable similarities in the thematic terminology employed across these 3 studies, although we conducted our analysis naïve to the existing literature so as not to influence our inductive approach. Such similarities offer some confirmation of the validity of our findings and emphasize the importance of these challenges.

## STUDY LIMITATIONS

Although the sample was representative in terms of established characteristics of mTBI and more representative in terms of age than previous research, it is nonetheless limited by the fact that participants were predominantly recruited from one major trauma center located in Cambridge, in the East of England. This may explain the high level of educational attainment (39% has postgraduate qualifications). Patients were recruited at different points in their recovery journey, from very early to subacute; this may have influenced perspectives. There is also a risk of social desirability bias, whereby participants provide responses that are more “socially acceptable” than their true opinions. The generalizability of findings from this single site, particularly those relating to the quality of care provided in the ED, is difficult to judge.

## CONCLUSIONS

We provide one of the first accounts of patients' experiences of mTBI from a health care management perspective from the United Kingdom. This extends previous findings and provides a broader oversight of problems that transcend the entire patient journey. Many of the problems identified were linked to information provision or communication, in one way or another. Those responsible for developing public health policies and NHS services should be mindful of these issues when they embark upon revision or improvements.

## Supplementary Material
