# Supplementary material for: The Experiences of People With Mild Traumatic Brain Injury Attending Emergency Departments in England
Source: J Head Trauma Rehabil. 2026 Apr 23;41(6):e601–8. doi: 10.1097/HTR.0000000000001168 (PMC13727030; doi:10.1097/HTR.0000000000001168)
Supplement: Supplementary file 1 [file jhtr-41-e601-s001.docx]

**Supplementary Figure 1**

***Reflections/memories of the hospital admission:***

a) Why were you admitted to hospital?
b) How did you get there?
c) What was the cause of your injury (What do you think led to your hospitalisation/ visit to the Emergency Department?)
d) What did you expect to happen when you got to hospital? What treatments did you expect? What did you expect to happen when you were discharged?
e) How close was what happened at the hospital in practice compared to what you expected?

***Transitions and Communication:***

a) What information and support did you receive about discharge and caring for yourself when you went home?
b) How were you followed up after hospitalisation? Who got in touch with you and why? Was it your GP, consultant, specialist nurse or other?
c) Did you feel that your follow up communications were ‘joined up’ between you and other healthcare professionals? Were you contacted by them or did you have to try and organise the follow up yourself?

***Impact:***

a) During your hospital visit did you feel that you were listened to and understood? Did you understand what the doctors and nurses were telling you?
b) When you were discharged from hospital did you understand the information/guidance that was provided to you? Were you able to ask questions?
c) What/who helped you after discharge? Were you with a family member at discharge that you were living with whom could help you at home?
d) Were you given any information about other sources of help after discharge such as charities which focus on brain injury? Did you use any of the other sources of help?
e) Were you told that you would have any follow up from the hospital services or your GP? Or were you told that you would need to contact these services only if you felt you had ongoing symptoms of the mTBI? Were the possible symptoms described to you so that you knew what to be aware of?
f) Did you feel confident about who you could contact if you felt you had ongoing symptoms of your mTBI?
g) What impact has your mTBI had on your daily life?
h) Have you had any issues with your memory or ability to concentrate? Do you feel your thinking has slowed? Are you struggling with your attention span?
i) Are you having any issues with your emotions or sleep? Are you more irritable or quick to get angry?
j) Has your mTBI had an impact on your social or family life? Are you finding it difficult to be with larger groups of people? Are you more irritable with family members? Are you finding your relationships more strained?
k) Have you had any physical effects/symptoms of your mTBI that are ongoing? These could include headaches, dizziness, blurred vision, neck pain, fatigue and/or sleep disturbance).

***Post hospital:***

a) What have you done/changed since the mTBI? Are there any changes that you have made to your daily activities? Do you feel that your level of independence has changed?
b) Have you contacted any healthcare professionals or other services after your mTBI? If you did make contact, whom did you contact and in what context?
c) Did you find that these services helped and if so, how?
d) If you did not find these services helpful, what would have made the service better for you?

***General perceptions:***

a) Overall, how do you feel your experience of your treatment and post hospital recovery has been? Was it a good or bad experience?
b) Did you feel that you had any choice in the way that events were unfolding?
c) How informed did you feel throughout your mTBI experience?
d) What elements of your experience could have been better?
e) Are there aspects of your recovery process that you feel could be improved as far as help and support is concerned? What elements do you feel are missing?
f) The Future:
a) What are your thoughts about how you might deal in the future with issues while recovering from your mTBI?
b) How would you describe your continued ability to manage daily activities at home?
c) Have you been able to manage any changes your needs since your mTBI?
d) Do you have any worries or concerns about these issues?

***The Ideal:***

a) What do you think would be the ideal care after mTBI, based on your experience?
b) What would make things easier or better for patients with mTBI, either in hospital or during your recovery?
c) Are there any aspects of your treatment or follow up that were better avoided or in need of review by healthcare professionals or other service providers?

**Supplementary Figure 2**

**Coding Cycle 1**

**Coding Cycle 2**

**Thematic Map - illustration**

**Supplementary Table 1**

| **Theme** | **Quotes in support of theme** |
| --- | --- |
| *Decisional Difficulty - Consulting Conundrum* | “I mean, I think the reason why I went to A&E was more to do with [my other disease] because you can have an [exacerbation] and some of those symptoms are very similar, like feeling sick and hot and those kind of things, and I just wanted to make sure” (IABA017)  how…how serious is this? Or is there any way I can test myself before I go and take up space and time in the hospital?” (IABA009)  “I have felt a little bit that I shouldn't be troubling the A&E because I should have gone back then [at initial injury].” (IABA036)  “I wasn't showing any sort of really immediate injuries.” (IABA025)  “Then a lot of people would think, oh it’s ridiculous, I’ve got a headache, why would I want to go to ED, you know, you can see they’re so busy, I’ve got a headache for god’s sake, I’ll just take some more paracetamol…somebody would go, oh just soldier on…” (IABA011)  “Yeah, I mean, I always feel guilty. You know, accessing any sort of health care, because I'm like, oh, do I really need it? “ (IABA025)  “Umm, because I truly believe in not trying to overload the hospital if you possibly can” (IABA009)  “you know, you can see they’re so busy, I’ve got a headache for god’s sake, I’ll just take some more paracetamol. (IABA011)  “The thing is you don't know the difference between being dazed [post mTBI] and drunk, really.” (IABA008)  “I think he had the possible diagnosis, although they've never confirmed it….of Parkinson’s” (IABA016) |
| *Decisional Difficulty – Limited alternatives* | “Before I went to A&E, I actually got in touch with the GP and the triage nurse rang and said ‘the GP said no point seeing you’. You need to go to A&E.” (IABA036)  “well, my son called 999 but that was not going anywhere so we, my daughter’s boyfriend got us an Uber to [the hospital].” IABA037 |
| Lacking Holism – narrow focus | “I mean, coming out of hospital, I was kind of cross with the way we've been dealt with….and felt very much that I was one in a million rather than I was me .” (IABA036) “[here is] a bed…’Can you just walk across to go here onto the bed?’, and I'm just like, “But I can't walk, you need to lift me over’, and then…’yeah, but surely you can just step off the bed and walk’…’no, I can’t!’” (IABA001)  “No, but did they do a full check of your body when, you know, all the way through? (IABA001)  “No...The only, the first time I knew about that bruise on my stomach was when I was being discharged and my sister was getting, helping me get dressed” (IABA001)  “And the other big thing we were asked was cause, I guess, come back from a rugby match oh, has he had any drinks? But and I hadn't actually, I've been very sensible” (IABA013)  “I think the third time what they did and how they treated him was absolutely right and was fine. And he got to the bottom of things and they didn't discharge him until they knew that he was able to cope.” (IABA016)  “they just took it as an old person falling. And you know, sort of let’s dress his wounds and, you know, take him home, you know, take him away from A&E.” (IABA016)  “[there was no] discussion to see where we are at and to get more information about the life circumstances. A&E just looks at your body and that's what they have to do. (IABA002) |
| Lacking Holism – Maze of Multiples | “The contrast between the doctor we saw at Hemel Hempstead on the, whatever day was, who said ‘No…you've got to have a CT scan’ and then the next one, he said ‘No, you only need an X-ray.’ And there's this complete sort of contrast between the two “ (IABA036)  “I think as well one of the other issues I had in um A&E was I was asking for pain relief and anti sickness, but I didn't get any of that the whole time I was there.” (IABA012)  “And then the CT woman was asked to wait to have some blood and I was explained the bloods were for a general check and before being given some anti-nauseous drugs that they need to check on my bloods. But at that time I didn't know what was up with the imaging. And it's only after they've taken the blood. That one of the doctors I saw on the path, not the consultant, but someone else and said, well, actually, you're good to go” (IABA002)  “They, eventually yeah, I think I was there probably four hours before they did a CT. And… I literally saw the doctor five minutes before I got discharged, that was it.” (IABA011)  “I would say within half an hour I went to speak to someone who, umm was uh, I a little bit matter of fact about it, but I think he was just giving to me what a consultant or somebody had told him that no, they can't find anything.” (IABA008)  “later they finally had a bed, that we then lifted her onto, and they had us in a cubicle and then they moved us into another cubicle and then there was another bed they needed to get us onto.” (IABA001)  “I felt a bit rushed, to be honest. Like they're trying to just get me in and out” (IABA005)  “but like GCS, it’s like the basic stuff, do you know where you are, can you, you know? That sort of nothing, and there was no baseline, so they wouldn’t have known if I’d deteriorated, you know?” (IABA011)  “the male Doctor who um did that well, he I think he was the one who asked for CT. He just kind of said that I don't think you need it, but we're gonna do it anyway.” (IABA012)  “So I went to A&E and I had the referral from the GP saying go to A&E and have a scan and I got to A&E and they were like well if you didn't actually hit your head, we're not sure you hit your head. Do you really need this? And it was just like I have taken an afternoon off of work. I have come all the way over here, the GP has asked they say they want to do this. Would you please just do this? And I that was the most frustrating part because I felt like I was having to argue to have something that someone had already just already decided would be a good idea.” (IABA025)  “I think somebody came past and said, “Oh what are you still sitting here for?” I said, “I think I’m waiting for a CT scan,” “Okay, I’ll chase it up,” and then within like half an hour I was off to CT” (IABA011)  “there was a point just after I got sat down in the ED that I actually felt nauseous, I didn’t vomit, but I felt nauseous, my sister had to go and find a vomit bowl for me. But nobody came to check and see if I was okay” (IABA011)  “Yeah, I think it just became quite confusing what was what.. it's it's all over the place.” (IABA017)  “Having seen the doctor and then being sent for X-ray, she then just kind of bumped into me in the waiting room as she was walking back out and said, oh, by the way, your X-rays clear so you can go now. And that was it. “ (IABA036)  “I would say within half an hour I went to speak to someone who, umm was uh, I a little bit matter of fact about it, but I think he was just giving to me what a consultant or somebody had told him that no, they can't find anything.” (IABA036)  “I was seen by 1/2 a dozen people in one form or another.” (IABA008)  No, they've at first they said they would [do a CT] and then…on review with the consultant said they wouldn't. They didn't think it was necessary.” (IABA009)  “My expectation was at least if I had to sit in a wait room that I’d be somewhere a little bit closer to somebody, or somebody would be coming up and telling, like asking me frequently if I’m okay. At the very least doing a set of neuro observations on me”. (IABA011)  “Well kind of just, he kind of just said that everything’s fine, your nose looks like wonky inside or something…He was just kind of quick to discharge me.” (IABA012)  “And there was no way I was going to do that in the publicity of a waiting room where, you know, everyone so bored that any doctor that talks to you, you're all earwigging and listening.” (IABA036)  “And I was in the emergency department and within a relatively short time within 30 to 40 minutes they sent me across to the urgent medical care where I thought they were just gonna either put a patch or put a stitch or something in the they basically sort of had a had a look made a couple of phone calls etcetera and I thought I was gonna get away fairly quickly, and then they said because you've hit your head and your on rivaroxaban, etcetera. You're gonna have to go back to the main emergency department and you'll be called for more checks.” (IABA014) |
| Lacking Holism –‘Deficient Discharge Dialogues’ | “I would have hoped for more [information] because I googled it and looked, you know, for myself, for my own peace of mind what I should know to try and help” (IABA016)  “As soon as she [the doctor] worked out that there were probably more important people than me, I was bustled out, you know… but I think, as I say, the key thing that she told me, which I wouldn't have realized, is that after effects could come considerably later” (IABA033)  “Yes, I was told I was told on my way out by the young doctor. Look, there's a QR code here. That's the website of the NHS, where you may find additional information, flash the QR code and you'll have the information there was like, OK. And to be fair, I read it, but there's not tons on there.” (IABA002)  “No, there was no paperwork, no next steps. No nothing. It was just you’re good to go and I remember thinking, OK, I'm good to go.” (IABA002)  “it was you’re good to go…and I understand there were very busy as always and there were a lot of people to be seen. But a sat down conversation 5 minutes somewhere would be useful, and something in writing summarizing the key points discussed or what to do if, yeah. (IABA002)  “Um I think that I did find it quite useful, but it would have been nice for like the doctor to kind of speak to me about it instead of reading the leaflet.” (IABA012)  “Right, okay, well you’re at high risk of getting concussion, are you living on your own?” “Yes, I am, but my sister’s going to stay with me today,” “That’s fine, just need to make sure you do X Y Z,” which was take pain relief when needed, keep your fluid intake up, stay off your mobile phone, stay off TV for the next few days, or try and reduce your screen time. If there’s anything concerning you come back to ED, and we can give you a QR code that you can scan to find out more information, which I didn’t get.” (IABA011)  “I'm am I thinking back through the process it it's a bit of a concern that I wasn't sent home with a leaflet of these are the things to look out for.” (IABA009)  “there wasn't any time because….they're asking you questions and you're just responding and then you know, you get no feedback you know, not even an information sheet. Nothing.” (IABA036)  “No, he didn't [describe probably mTBI symptoms]…and in the days that followed I did begin to worry about whether I had a post-concussion syndrome because I was tired and, you know, sleepy and, but that, after about 5 days that wore off. “(IABA039)  “And I thought that was a lack, I needed, you know, if I'd been Joe Blow off the street, he should have said, "You will, if you are sick, if you lose consciousness, if you're feeling not part of the world, please go and see your GP or come back to A&E". (IABA039)  “Just verbal advice…I didn't get any [written information]. Mind you, it was an incredibly busy night, as you might imagine. “ (IABA039) |
| Lacking Holism - Follow-up, A Scarce Commodity’ | “And I say afterwards, there's definitely, I guess, no contact at all. So probably would have been better to have some more [discharge] guidance.” (IABA005)  But even just a call, even from a nurse, it doesn't have to be GP….A few days after a few a couple of weeks after to say where are you at that would be useful.” (IABA002)  “…but there was no mention of saying, like, we’d prefer that if you go to your GP in a couple of weeks’ time, just to make sure everything’s fine, or that the GP will be notified of your admission here, so there was nothing like that.” (IABA011)  “Umm yeah, it would have been nice to have something more formal to help you, I don’t know, maybe get back into life instead of just guessing.” (IABA005)  “but the body functions very differently depending on the environment we evolve in, the constraints we have upon ourselves. So to me, just a quick check 48 hours after to see if everything's OK, and if we need to go back and be seen or have additional support and yeah.” (IABA002)  “After I left the hospital, obviously I had no other contact whatsoever…So I suppose the onus on it was on me [to secure follow-up].” (IABA008)  “No, not at all. It was all quite disjointed really and. I think the answer that I got was well, if you….do feel that you're at risk at all, you're still not feeling too good, then you should go to A&E.” (IABA017)  “Umm, I think for example they did have a follow-up phone call couple days afterwards…That would actually really make them feel like actually they've been properly cared for.” (IABA013)  “I would have liked it in my situation of being, umm, very privileged and everything, but even I would have liked it if I've had a message or something how to say look, this is what you if you're still experiencing it, don't be afraid to contact.” (IABA008)  “it’s like we always say, somebody giving you permission to go to ED isn’t it, and but that they would have been pretty good [follow-up] I think, to be honest with you” (IABA011)  “it was almost like, okay, that’s it, just sort yourself out.” (IABA011)  In the part of Australia where I trained, I'd have had a second scan to make sure everything was alright, rather than just the first one in the hospital, but that's, that's pretty par for the course in the current [climate]“ IABA039  “Then he said, "You can go home", but, of course, he said what's always said, "Well, if you have any problems, get in touch with us", but they don't tell you who to get in touch” (IABA039) |
| Uncertainty Central - ‘The Saliency of Knowledge’ | “so I did try to be good, I tried to have siestas and I said, ‘no gardening’, which was a bit frustrating because the garden's tramping on us…nobody really explained that, but…I realized that doing jobs that were involved in bending forward are not so comfy.” (IABA024)  “And so it's not my first concussion….And knowing that it's about patience now…. I just need to take it easy and that's why, although I'm a very sporty person, actually today we are almost two months after…I have not returned to exercise.” (IABA002)  “No. And I mean, I started looking online as well. Brain training and doing, doing things really to help myself…I think it did help a bit. It was self-empowerment as well. So yeah, I think it did, although I struggled a bit with it and found that at the time it made me even feel a bit more tired. But I was doing meditation and relaxing as much as I could.” (IABA017)  “but I kind of used some of the information I got off the internet, to inform me how I would go back to work…having not had a significant like bang to my head before, I just wanted to make sure that what I was feeling was relatively normal. And like I was looking at it thinking, okay, yeah, that’s what I’m getting there, that’s fine, okay, alright, so it might take a little while for that to figure out, right okay, I’m good, I’m good.” (IABA011)  “I think my expectation is that concussion can last a long time and there's no quick fix to it and there's no medication that I can use to get rid of it.” (IABA036)  “But also just the amount of activity that I should have been doing just, you know…how much activity, how much physical activity is sensible for a brain that's been shaken around….and at that point, you're just living by the mantra of, well, I'll do what I feel is right and my body will tell me which potential dangerous..” (IABA036)  “So I, I was happy with my post-concussion aftercare, if you like, but it was all self-directed, it wasn't part of the protocol. “ (IABA039)  “but I also think seeing the same person is important, because what's, you're actually, underlying it all, worried, frightened, what have I done to myself, and by seeing a competent professional, who's also kind and talks to you easily, really makes that aftercare effective.” (IABA039) |
| Uncertainty Central - ‘A License to Recover’ | “the GP, though, suggested that I should take a couple of weeks signed off work… but indeed if I had not been to see my GP, thinking of other people, then I would not have been offered the opportunity for time of work, yeah.” (IABA002)  ““Umm, so basically [the GP] told me to take, to have plenty of rest, and if I can, really to have a follow up in another month. That was the first advice. Yeah, I also had sensitivity to light, umm and noise and he just said that it would improve you know just try and be mindful and rest and not do too much really.” (IABA017)  “I decided to approach the GP because I felt surprised at how forgetful and tired I was…. And I just wanted that reassurance and guidance.” (IABA002)  “And I feel like, oh, my God, what's gonna happen in the next day [at work], after on Thursday, I called the GP. The GP saw the statement from Addenbrooke’s and she put me two weeks off” (IABA013)  “I last week I went to Specsavers….I know there's nothing else going on and I left sort of being a bit reassured, other than the fact that I've never been ill for more than two days at a time, so being affected by something for two or three weeks is a bit of an eye opener or not.” (IABA008) |
| Uncertainty Central - Sensitized Spaces’ | “So the next day I was in work…Umm, but after a few days I remember telling my boss who's very lovely, and she let me take it at the pace I wanted with my return to work.” (IABA002)  “[my husband] looking after the toddler as well, which I'm grateful for, otherwise [it] would have been very much, a very difficult few days” (IABA002)  “But it was, you know, left up to me to go back to work, so I thought, well I’ll come back to work, and see how I go, that’s basically what it was.” (IABA011)  “...so I kind of like eased myself back in…but there was no, yes, it’s I’m now ready, it’s kind of like, oh, I think I’ll see how it goes, it’s like how long do I take off, you know?” (IABA011)  “I thought, well, I've got [to do] a bit of work I need to pay the bills and there's no really chance of changing the way my lifestyle is at the moment.” (IABA017)  “And I came back [home after one month] feeling a lot better. And I think part of that was that I didn't have to parent….And so and I, well, I only had to deal with myself for like executive function and things like that.” (IABA025)  “Yeah, I was making a lot more lists and I was having one of my colleagues take notes in meetings…. I wasn't confident in my ability to take notes and continue to parse what was going on in the meeting.” (IABA025)  “I told him in the next day I'm gonna be off sick [for a] couple of days because I'm not OK…. then after the second day he starts calling me and telling me you have to give me a call. I gave him a call, [he] even made me cry on the phone.” (IABA031)  “And then I said, “Oh I’ve only been paid five and a half hours?” she [manager] said, “Why?”, I said, “Because when I texted the other senior nursing sister, my incident happened at 11.30, and I got wheeled off to ED at 12. So she’s put me down as being…[not at] work for that half an hour, it’s like, oh for god’s sake, it’s just, oh my god.” (IABA011)  “But also just the amount of activity that I should have been doing just, you know…how much activity, how much physical activity is sensible for a brain that's been shaken around….and at that point, you're just living by the mantra of, well, I'll do what I feel is right and my body will tell me which potential dangerous.” (IABA036)  “I just rang my boss and had a chat because it was so much easier because I was quite stressed because the contract that I have is that I don’t get sick pay so I would just get Statutory Sick Pay.” (IABA037)  “My other jobs are teaching music and proofreading which I do to supplement what I do and I can’t do any of it, so.,,,No. I can’t cope if more than one person’s talking at a time to me and I used to always have music on and I don’t seem to be able to filter anything out, so I’m actually listening to the music rather than background so that’s my whole brain tied up is what it feels like. It’s very odd.” (IABA037)  “And I’ve actually got the kids to only message me if possible unless it’s an emergency between 7 and 8 in the morning and in the evening so that I’m not constantly looking at it when it beeps, I just save everything up, if that makes sense.” (IABA037  “Yeah, so I’d done a week of work, I got sent to the urgent treatment bit, Clinic 9, the GP, I saw a GP in there who was very nice, did all the same tests, felt my head, signed me off for another 3 weeks, said, “I think you’ve got post-concussion syndrome which is just concussion longer,” and just to take it easy and hopefully it’ll get better.” (IABA037)  “so I then looked up post-concussion syndrome on it [NHS website] and got thoroughly depressed because basically my job is incompatible with what I’ve got.” (IABA037)  “I know, and I just thought, ‘Oh terrific,’ but yeah, you know, I just feel like there should be something I can do that might help, I don’t know if there’s a way of, I mean phase back into work is a very open-ended statement, I’m coming to the conclusion that I actually need to work when I feel good, not just do short shifts when I am scheduled to, if that makes sense.” (IABA037) |
